# Adapted Physical Activity Protocol for Lower Limb Functional and Strength Recovery in a Young Athlete with Cutaneous Melanoma: Feasibility and Efficacy during COVID-19 Pandemic

**DOI:** 10.3390/ijerph19159590

**Published:** 2022-08-04

**Authors:** Giuditta Carretti, Daniela Mirandola, Sara Germano, Mirko Manetti, Mirca Marini

**Affiliations:** Department of Experimental and Clinical Medicine, Section of Anatomy and Histology, University of Florence, 50134 Florence, Italy

**Keywords:** adapted physical activity, melanoma, skin cancer, health-related outcomes, cancer survivor, quality of life, COVID-19

## Abstract

Adapted physical activity (APA) can improve psychophysical wellbeing and quality of life (QoL) in cancer survivors, a vulnerable population requiring a global management, especially during the recent pandemic. On this basis, we investigated for the first time the impact of a tailored APA intervention on a melanoma-affected 18-year-old female athlete to counteract treatment sequelae and promote lower limb functional and strength recovery. Patient was evaluated at baseline and post-protocol by a test battery focusing on mobility, muscle strength measured by dynamometry, and lower limb girths assessed at specific anatomical points. Moreover, health-related QoL, depression/anxiety, psychological distress and pain intensity were evaluated by Functional Assessment of Cancer Therapy–Melanoma (FACT-M), Hospital Anxiety and Depression Scale (HADS), distress thermometer, and numerical rating scale (NRS) questionnaires, respectively. An almost doubled up increase in lower limb strength, along with hip mobility improvement, and post-surgical edema and pain reduction were observed following the protocol. Concerning the QoL assessment, a moderate post-intervention improvement in physical and emotional wellbeing was detected, while depression state worsened though remaining within the normality range. Our findings show that a specialist-supervised structured APA protocol based on a patient-centered multidisciplinary approach may represent an effective strategy to recover functional and psychophysical efficiency, thus promoting a quick return to daily life activities and offering a concrete chance of resuming competitive sport practice.

## 1. Introduction

Growing and recent evidence of COVID-19’s psychological impact highlighted an increase in depressive and anxiety symptoms along with impaired sleep quality in the general population, and mostly in women, students, and younger adults, compared to pre-pandemic times [1]. The World Health Organization preventive measures [2], by limiting social interaction, promoting self-isolation and temporary closing sports facilities, heavily affected daily life and negatively impacted mental/physical health and training habits, hence raising sedentary behaviors [3]. Since regular practice of physical activity is a well-known crucial protective factor for overall health [4], such a global training reduction is of major concern, especially during challenging time as in a pandemic [5,6]. Mental health is a peculiar component of public health associated with a reduced risk of various chronic diseases, premature morbidity, and functional decline [7]. Social distancing policy damagingly affected general mental health and well-being, and particularly forced athletes to face a difficult challenge concerning their habits and sportive identities. Abruptly ending sport practice may become an identity threat for this target population, thus making the athlete experience a sense of loss and further increasing depression risk [8]. Moreover, research on muscle adaptations to unloading reported that mass and structure changes in lower limb muscles can occur even after a short-term detraining, and such disuse atrophy may even be accentuated in competitive athletes, thus affecting muscular contractile capacity and injury risk [9].

Another target population deeply influenced by the COVID-19 pandemic has been the oncological. Cancer is a complex and multidimensional disease usually difficult to bear and manage in normal circumstances, and which may even become overwhelming in a stressful context such as the recent pandemic [10,11]. Among cancer patients, the clinicodemographic characteristic showing a significant association with higher psychophysical distress vulnerability is female gender [12,13]. Particularly, melanoma-affected patients experience a consistent anxiety and distress level strongly related to body image alteration caused by surgical scarring and potential secondary lymphedema onset [14,15], both threatening subject self-esteem, autonomy, and quality of life (QoL). These common surgery sequelae are frequently accompanied by tiredness, mobility reduction, discomfort, and pain, thus forcing patients to progressively quit daily life activities such as housework, hobbies, and social interaction with an adverse impact on their mood [16]. Despite the high incidence of surgical scars, surprisingly, little is still known about their impact on patient lives, though scientific research suggests that it may be related to both physical and psychosocial effects [17]. Concerning the emotional dimension, scars are perceived as a constant reminder of their conducive event that often hides traumatic memories. Many subjects, especially young women, following surgery for melanoma, face difficulties interacting with opposite sex individuals due to their physical appearance. In addition, open air leisure activities are often reported as distressing since they require body and scar exposure to the sun [17]. Regarding the physical and functional dimension, post-operative complications, such as secondary lymphedema onset occur in most patients undergoing melanoma-related inguinal lymph node dissection [18]. This condition, together with skin grafting necessity and wound complications, frequently leads to prolonged hospitalization and consequently delayed return to normality, which drastically reduces physical functionality and efficiency [19,20]. Even if in mild stages, the negative impact of lower limb secondary lymphedema on health-related QoL deserves particular attention in the global management of melanoma patients [21]. Given its wide surface and its superficial location, skin is one of the largest human body organs and the most environment exposed. Deeply attached to the subcutaneous fascial system, it provides bodily continuity and, being afferently/efferently connected to all the anatomical systems, influences the entire body homeostasis, including emotions [22]. It can be intuitively assumed that, in case of dermis and fascia affected by scars, their structure and function, intended for interaction with the external and internal environment, are strongly depleted [23]. The direct consequence is an ample symptomatology affecting not only the area where scars are located, but the whole-body functionality [22,24]. Regardless of surgical procedure applied, when scar adhesions affect the ankle, this results in tibio-tarsal joint mobility reduction, afferents/efferents alteration, as well as coordination and gait pattern dysfunctions, along with an incorrect load distribution, consequently affecting postural control, proprioception, and balance [25,26]. In addition, those postural adjustment alterations and the related increase in antigravity muscles tension lead to dysfunctions in body areas less capable of compliance and ankle dynamic-connected, such as the thoracolumbar fascia, with the consequent onset of back and shoulder painful symptomatology [27]. Since the lumbodorsal spine is closely connected to the diaphragm, its stiffening may generate respiratory malfunctions, which in turn affect emotional regulation and stress management, hence feeding a vicious circle [28].

Based on the aforementioned skin cancer-related sequelae and their management, especially difficult during a pandemic, and considering the well-known positive role of physical activity on health and psychophysical well-being [29,30,31], here we investigated for the first time the potential benefits of a tailored adapted physical activity (APA) protocol addressed to a young female athlete affected by melanoma and its scarring complications.

## 2. Materials and Methods

### 2.1. Case Description

A 18-year-old female volleyball player was referred to the Cancer Rehabilitation Center (Ce.Ri.On) in Florence for cancer-related follow-up management. Specifically, the Ce.Ri.On provides a structured multidisciplinary pathway organized in progressive stages involving different professional figures (i.e., oncologist, physician, physiotherapist, psychologist, dietitian, and APA specialist) for oncological patient global recovery.

**Figure 1:**
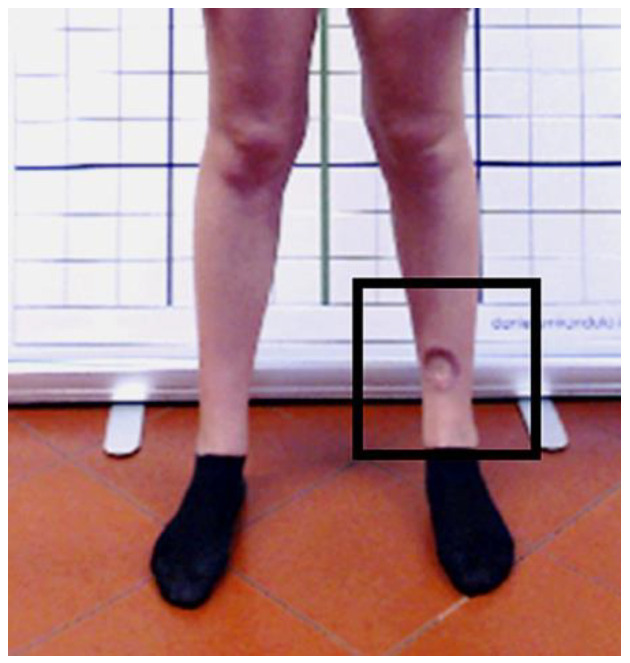
Post-surgical scarring at the left anterior tibia after melanoma resection (black squared frame).

Concerning the young athlete’s clinical history, in October 2019 she underwent biopsy for a cutaneous suspected erythematous nodule clinically localized at the left anterior tibia. Histological evaluation showed the presence of proliferating fusiform melanocytes. Therefore, in December 2019, she underwent surgery consisting of a wide local excision, and the subsequent histopathologic report revealed a desmoplastic melanoma. Patient was also routinely monitored by computed tomography scans of head, chest, and abdomen showing no distant metastases. However, in May 2020 further surgery was needed for local recurrence with biopsy of two sentinel lymph nodes. Due to the surgery’s high invasiveness, a skin graft was required, thus resulting in significant scarring outcomes (Figure 1).

In addition, since the lymph nodes were pathologically involved, patient underwent inguinal lymphadenectomy removing seven lymph nodes at left, and the Cloquet’s node in July 2020. Subsequently, she was treated with immune therapy (i.e., nivolumab) for 12 months. Due to cancer diagnosis and treatment sequelae, the young athlete was forced to stop volleyball practice after an eight-year career. Her total training per week was about 12 h consisting of 6 h of volleyball-specific training and 2 h of fitness center weight training. In addition, two weekly matches of about 2 h each were scheduled for the C league which she belonged to. In September 2020, the Ce.Ri.On rehabilitation physician diagnosed a reduced tactile sensitivity referred to the left inguinal region and keloid presence, as well as atrophic scar on left anterior tibia with absence of symptoms, left ankle mild post-surgical edema, and homolateral hip referred pain. The physician prescribed lymphatic drainage treatment (i.e., seven sessions) for left lower limb. Then, in October 2020, on the basis that there was no medical contraindication, participation in a structured APA pathway was recommended. The study was carried out following the rules of the Declaration of Helsinki. Since the patient was referred to the Ce.Ri.On oncological rehabilitation pathway, no ethics committee was needed for this publication, and the Italian Protection of Personal Data Law (D. Lgs. n. 196/2003) was complied. A signed informed consent form was obtained from the participant.

At baseline and after ending the structured APA intervention, the subject was examined by the APA specialist through a functional test battery such as active range of motion (ROM) test, evaluated by miniaturized inertial sensor (Moover, Sensor Medica, Montecelio, Rome, Italy) [32], to assess joint mobility (expressed in degrees) of different anatomical regions. Specifically, trunk flexion (normal reference range 0–50), extension (normal reference range 0–30), lateral inclination (normal reference range 0–45), and rotation (normal reference range 0–50) were evaluated, as well as upper limb flexion (normal reference range 0–180), extension (normal reference range 0–45), abduction (normal reference range 0–180), and adduction (normal reference range 0–45) [33,34]. Hip flexion (normal reference range 0–120), extension (normal reference range 0–30), abduction (normal reference range 0–45), adduction (normal reference range 0–45), and internal and external rotation (normal reference range 0–45) ROM were also assessed [35]. In addition, muscle strength measured in units of kilogram by dynamometry was considered as a further lower limb functionality parameter. Isometric strength, exerted against a resistance manually performed by APA specialist handling the specific digital tool (Activforce Digital Handheld Dynamometer, San Diego, CA, USA), was evaluated using the method and customizable muscle testing protocols recommended by the manufacturer, and recorded via the ActivForce app [36]. Precisely, hip flexion/extension and abduction/adduction, knee extension, and ankle plantar flexion, dorsiflexion, inversion, and eversion were tested. Lastly, the circumference of both lower limbs was also assessed by measurements at several anatomical points from foot to hip with a thin, flexible tape [37]. A ≥ 3 cm girth difference at any measurement point between the affected and the unaffected lower limb was considered indicative of moderate/severe lymphedema.

Moreover, the subject filled out several questionnaires provided by the APA specialist, such as the Functional Assessment of Cancer Therapy–Melanoma (FACT-M) to evaluate health-related QoL [38,39], the Hospital Anxiety and Depression Scale (HADS) to assess anxiety and depression [32], the distress thermometer to rate psychological distress (PD) [32], and the numerical rating scale (NRS) to quantify back and lower limb pain intensity [32,37]. Specifically, the FACT-M questionnaire was proposed as a reliable and valid instrument that can be used for QoL assessment of patients with melanoma in clinical trials [40]. FACT-M derives from the generic FACT-General (FACT-G), which is applied for all cancer types and consists of 27 items divided into four domains: Physical Well-Being (PWB, 7-items); Social and family Well-Being (SWB, 7-items); Emotional Well-Being (EWB, 6-items); and Functional Well-Being (FWB, 7-items) [38]. The items, which are phrased in the first person, refer to health-related QoL during the past week. The rating scale runs from 0 ’not at all’ to 4 ’very much’, with a total score ranging from 0 to 108, where a higher score indicates greater QoL. The FACT-M questionnaire contains 24 additional questions on melanoma specific symptoms consisting of 51 items: 27 items comprising the FACT-G subscale; 16-item Melanoma Subscale (MS, total score ranging from 0 to 64); and 8-item Melanoma Surgery Scale (MSS, ranging from 0 to 32). The Trial Outcome Index (TOI) equals the sum of the PWB, FWB, MS and MSS subscales. Melanoma Combined Scale (MCS) is calculated as the sum of MS and MSS [38]. To detect patient anxiety and depression state, the HADS self-administered questionnaire, composed of two 7-item scales, was also used [32,41], as well as the distress thermometer to assess PD [32]. Concerning HADS, scoring for each item ranges from zero to three, with a total subscale score of ≥8 points out of a possible 21 denoting considerable symptoms of anxiety or depression (i.e., 0–7 = normal, 8–10 = borderline abnormal, and 11–21 = abnormal) [41]. The distress thermometer is a visual graphic scale consisting of 11 points with a range from 0 (no distress) to 10 (extreme distress). Subject is required to indicate the personal level of distress over the course of the week prior to completing the questionnaire. Finally, the NRS evaluates pain intensity on a 0–10 scale (0 = no pain, 10 = worst imaginable pain) [32,37].

### 2.2. Structured Physical Activity Protocol

**Figure 2:**
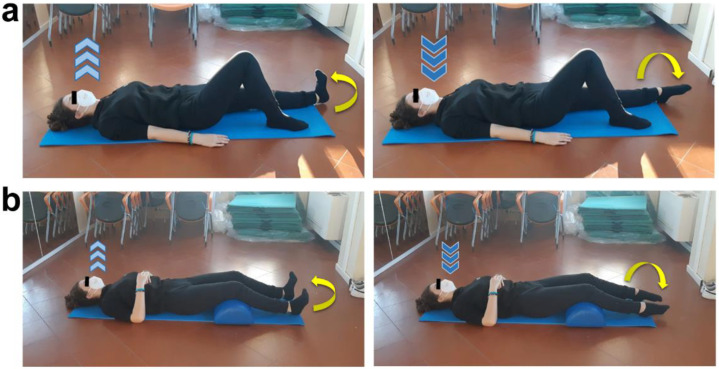
Tibialis anterior strengthening exercises. (**a**) Supine position, resting leg flexed and working one extended, arms laying by body sides with hand palms down. Inhale, exhale (light blue arrows) while performing a dorsiflexion of the straight leg foot (pointing up yellow curved arrow), and then inhale (dark blue arrows) while performing a plantar flexion of the same foot (pointing down yellow curved arrow). Three sets of ten total movements (dorsi/plantar flexion) for each foot, 30 s of recovery. (**b**) Supine position, rest popliteal fossa of both legs on a semi-cylindrical cushion, bend arms and place hands on chest. Inhale, exhale (light blue arrows) while performing a dorsiflexion of both feet (pointing up yellow curved arrow), and then inhale (dark blue arrows) while performing a plantar flexion (pointing down yellow curved arrow). Three sets of one minute and a half each, 20 s of recovery.

**Figure 3:**
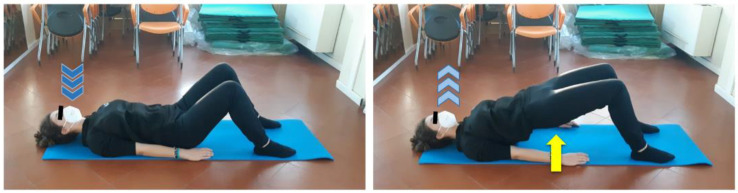
Glutes strengthening and hip mobility exercise. Supine position, flex both legs, set feet hip-width apart close to glutes, arms laying by body sides with hand palms down. Inhale (dark blue arrows), exhale (light blue arrows) while lifting glutes from the floor performing a bridge (yellow arrow) until reaching a complete hip extension. Keep this position for a few seconds, then slowly get back to the starting position. Focus on core stability, unload body weight on feet, arms and shoulders and keep lumbar spine in neutral position while performing the bridge. Three sets of one minute and a half each, 30 s of recovery.

The APA protocol was tailor conceived and planned by an exercise specialist in the oncological field based on the functional, motor, and joint mobility deficits detected during baseline evaluation and considering relevant preventive measures related to the COVID-19 pandemic. Specifically, the intervention lasted six weeks (from October to December 2020) and was organized in three 1-h sessions per week, one performed at Ce.Ri.On supervised by the APA specialist, and the other two self-led at home following the detailed exercise card also drawn up by the specialist. Since the young female athlete practiced competitive volley before receiving the oncological diagnosis, and post-surgical scarring and immobilization caused strength, functionality, and muscle trophism loss in her left lower limb, the main objective of the protocol was the gradual recovery of those skills. Moreover, the purpose-designed workout schedule also aimed to reduce lower limb mild secondary lymphedema and to counteract hip pain, thus intending to improve perceived QoL. In order to achieve those goals without any physical overload or postural compensation, first sessions were spent teaching subject to perceive, perform, and properly match movements and breathing. Afterwards, bodyweight customized exercises to strengthen specific muscles involved in lower limb and hip functionality, such as tibialis anterior (Figure 2), quadriceps, glutes (Figure 3), adductors, and abductors, were introduced.

**Figure 4:**
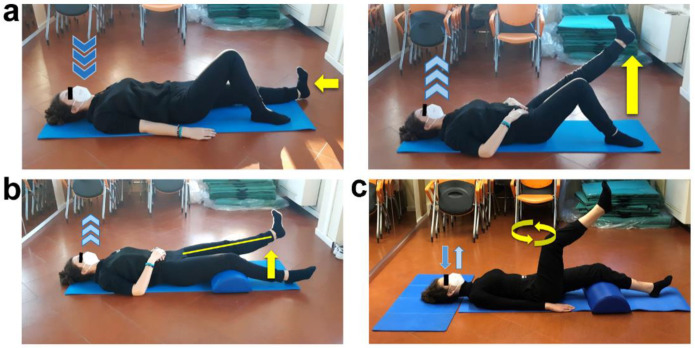
Quadriceps strengthening and hip rotation exercises in supine decubitus position. (**a**) Resting leg flexed and working one extended, arms laying by body sides with hand palms down. Inhale (dark blue arrows) while performing a dorsiflexion of working leg foot (horizontal yellow arrow), then exhale (light blue arrows) while lifting the extended leg upward (vertical yellow arrow) until knee reaches the height of contralateral one. Inhale and slowly get back to the starting position. Three sets of ten repetitions for each lower limb, 30 s of recovery. (**b**) Rest popliteal fossa of both legs on a semi-cylindrical cushion, bend arms and place hands on abdomen. Inhale, exhale (light blue arrows) while performing a leg extension (straight yellow line and vertical yellow arrow) with dorsiflexed foot. Keep this position for a few seconds, then inhale and slowly get back to the starting position. Three sets of one minute and a half for each lower limb, 30 s of recovery. (**c**) Arms laying by body sides with hand palms down, rest popliteal fossa of one leg on a semi-cylindrical cushion, flex and lift up the other leg until forming a 90° angle between thigh and trunk. Physiologically breathing (light and dark blue arrows), imagine drawing small circles by intra/extra rotating the lifted knee (curved yellow arrows). Three sets of 30 s for each lower limb, 30 s of recovery.

**Figure 5:**
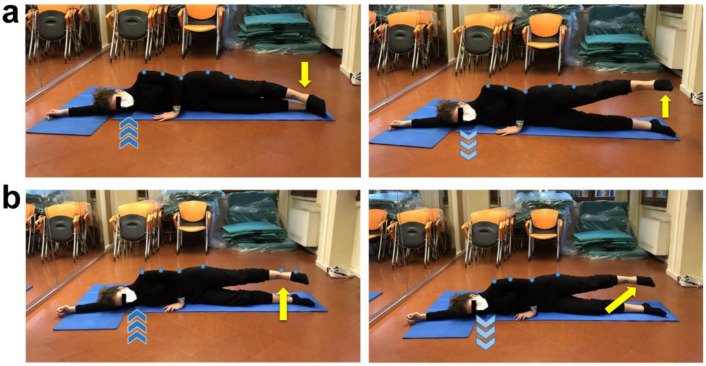
Glutes, abductors, and adductors strengthening exercises in lateral decubitus position. (**a**) Whole body straight in lateral decubitus position (light blue dots) and dorsiflexed feet together (pointing down yellow arrow), keep floor arm straight upward and rest head on it, bend top arm and place hand on the floor in front of the abdomen. Inhale (dark blue arrows) then exhale (light blue arrows) while abducting the top extended lower limb (pointing up yellow arrow). Inhale and get back to starting position. Three sets of 45 s for each lower limb, 30 s of recovery. (**b**) Repeat the previously described exercise adding an abducted lower limb extension on sagittal plane (diagonal yellow arrow), keeping lumbar spine in neutral position. Three sets of 45 s for each lower limb, 30 s of recovery.

**Figure 6:**
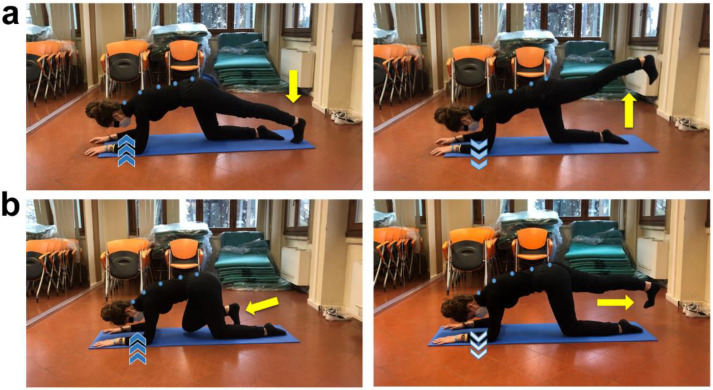
Lower limb extension and flexion in quadruped position. (**a**) Quadruped position, neutral spine attitude (light blue dots), place both elbows shoulder-width apart on the floor, keep the ground knee right under the homolateral hip and straighten backward the contralateral lower limb with dorsiflexed foot (pointing down yellow arrow). Inhale (dark blue arrows), then exhale (light blue arrows) while extending the straight lower limb on sagittal plane (pointing up yellow arrow). Three sets of 45 s for each lower limb, 15 s of recovery. (**b**) Repeat the previously described exercise performing a flexion of the other lower limb on sagittal plane by bringing the knee closer to chest with dorsiflexed homolateral foot (short yellow arrow), and then extend the same lower limb backward (diagonal yellow arrow). Three sets of 45 s for each lower limb, 15 s of recovery.

**Figure 7:**
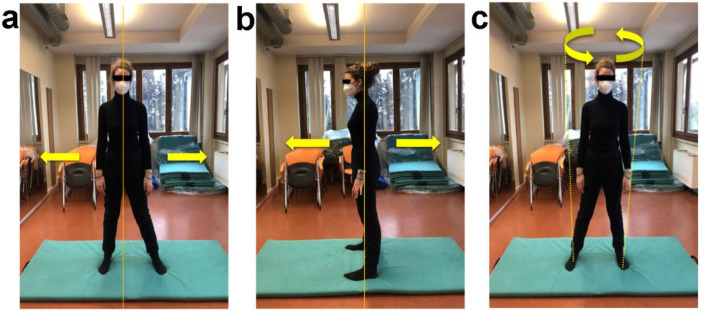
Postural sway on frontal, sagittal and transverse plane in orthostatic position. (**a**) Orthostatic position on a soft mat, feet hip-width apart, arms laying down on body sides. Keeping trunk aligned, shift bodyweight from one foot to the other on frontal plane (horizontal yellow arrows) without losing the ground contact. (**b**) Repeat the previously described exercise shifting bodyweight from forefeet to heels on sagittal plane (horizontal yellow arrows). (**c**) Combine the previously described exercises performing body circular sway on transverse plane (curved yellow arrows). Perform for one minute on each plane. No recovery.

Workout load was progressively increased by changing exercise body position, starting from supine decubitus (Figure 4), then lateral decubitus (Figure 5), and finally performing them in quadruped (Figure 6) and orthostatic (Figure 7) positions. 

In addition, even if strength and functionality recovery was particularly focused on left lower limb, the whole adapted workout program was designed as a symmetric total body training, thus recalling and supporting her sport competitive mindset and specific habits. This methodological approach promoted subject adherence to the protocol, both during supervised and home-based sessions, and granted a global and multidimensional management, therefore paying attention not only to physical health but also to the emotional/motivational component and the special needs of a young athlete facing an oncological path during a pandemic.

## 3. Results

Data concerning trunk and upper and lower limb ROM evaluations at baseline and post-APA are shown in Table 1. In particular, overall ROM values of trunk, including those rotation-related already greater than the normal reference range at baseline, increased after the structured APA intervention. Similarly, a general improvement in shoulder–arm mobility was observed (Table 1). Furthermore, hip ROM showed an evident improvement following the protocol (Table 1). Extension values mildly decreased, though remaining higher than the normal reference range.

Lower limb muscle strength data are provided in Table 2. A significant improvement was observed at the end of the APA protocol compared to baseline. Noteworthy, many values, especially those related to the left lower limb, showed an almost doubled strength increase (Table 2).

Table 3 shows lower limb circumference measurements acquired at specific anatomical landmarks. The subject presented a mild lower limb lymphedema at baseline. Although some differences between right and left lower limb girth increased after APA protocol, a relevant circumference decrease, especially referring to specific anatomical districts, was detected on both sides (Table 3). Of note, the evident left side post-surgical edema was reduced. Similarly, a disappearance of surgical left lower limb pain was also observed comparing baseline and post-APA (3 and 0, respectively). With regard to other anatomical districts, no initial pain was reported, and pain did not arise after APA (data not shown).

In addition, concerning QoL assessed by FACT-M, both physical and emotional wellbeing components, as well as the total score, showed a post-protocol improvement compared to the baseline (Table 4). In particular, the physical wellbeing component increased from 27 to 28 points, thus reaching the maximum subscale score (Table 4) [38]. Furthermore, whereas the anxiety component of the HADS questionnaire unchanged, the depression state worsened of four points post-APA intervention though remaining within the 0–7 normality range (Table 4) [41]. Finally, no difference between baseline and post-APA was detected for the distress thermometer, thus confirming PD absence (Table 4).

## 4. Discussion

Cancer survivors are an at-risk population that needs a multidimensional approach and management, especially in challenging times such as a pandemic [42]. Given the growing survival rate and the several oncological treatment-related psychophysical and functional sequelae impact on patient life [43,44,45], currently, the quality benchmark of an intervention on this target population is widely recognized in a patient-centered and one-to-one approach, as well as in a perceived QoL increase [16]. On this basis and fully respecting COVID-19 preventive measures, we designed our APA protocol as a mixed supervised and self-administered session program thus focusing on subject specific needs, promoting her autonomy, self-efficacy, and body consciousness, and granting training environment safety [46]. Moreover, as previously carried out and described [32,37,43], we aimed to design an easily reproducible protocol in order to promote a concrete APA application in oncological patient management. The obtained results showed that a tailored physical activity intervention, conceived as a symmetric total body training, may improve not only the surgical lower limb mobility and functionality but even the overall one, particularly addressing upper limb and back, and hence positively affecting many daily life activities-related motor patterns. Concerning our main objective, consisting in left lower limb strength and functionality improvement, we observed an almost doubled strength increase along with a reduction in lymphedema volume and an enhancement of left hip mobility. All these aforesaid results concretely contributed to recover a balanced load distribution and counteract postural dysfunction development. Of note, the surgical lower limb perceived pain decreased, which might have played a key-role in the observed improvement of physical and emotional QoL dimensions. Such a result, although limited in term of point increase on the specific subscales, may represent an encouraging cue to highlight, especially considering that our subject, being a young woman, a student, an oncological patient, and a competitive athlete, fell under all the socio-demographical categories most negatively impacted by the pandemic. Noteworthy, the aforementioned characteristics are well-demonstrated to be strongly related to depression and anxiety symptoms development [1,10,11,12,13]. In particular, concerning the depression state worsening, we could also hypothesize that individual session training, even though necessary to more quickly achieve the main goals and to minimize COVID-19 contagion risk, represents a methodological approach not easily accepted and appreciated by a young team sport athlete [8]. Moreover, despite the remarkable physical recovery, the missing COVID-related resumption of competitive sport practice might have played a crucial role on her mood at the moment of filling out post-protocol questionnaires [9]. Nevertheless, we should consider that, though worsening post-APA intervention, the depression state referred by our young athlete remained within the normality range [41]. The main limitations of our study regard the missing anxiety decrease, probably related both to the short-lasting protocol and the pandemic frame in which it was performed, and its case report-feature. From a future perspective, a subsequent introduction of collective APA sessions should be considered to better counteract anxiety and depression, especially in young subjects and athletes. Furthermore, a follow-up could be useful to monitor the long-term benefits of our APA protocol, not only from a post-oncological but also from a sport performative viewpoint. As far as the COVID-19 pandemic impact on athletes is concerned, it is noteworthy that the majority of published studies are related to post-infection recovery and usually do not provide any detailed exercise schedule [47,48,49]. In contrast, to the best of our knowledge, the present study is the first addressing the feasibility and efficacy of a structured APA protocol applied to a young athlete who did not contract SARS-CoV-2 but was undergoing cancer recovery in the context of pandemic limitations. Despite the growing melanoma incidence [50,51], there is an evident lack of literature addressing this target population’s complex needs, and in such a context we are confident that our study could provide an innovative and effective detailed methodological approach to apply and be deepened in further large-scale investigations.

## 5. Conclusions

A structured and planned APA protocol, tailored to the individual patient needs and supervised by a qualified APA specialist, may represent an effective strategy to improve lower limb functionality and strength in a young athlete with severe sequelae due to cutaneous melanoma treatments. Our encouraging findings confirm that a multidisciplinary management of the oncological patient is recommended to achieve the best functional, physical, and psychological outcomes [32,37,43]. When peculiarly addressed to an athlete, our methodological approach can promote a quick return to daily life activities, thus providing a concrete chance of getting back to the specific sport competitive practice.

## Figures and Tables

**Table 1:** Active range of motion test scores at baseline and post-adapted physical activity protocol.

Variables	Baseline	Post-APA
**ROM trunk**, degrees		
Flexion	38.6	44.7
Extension	17	17
Left lateral inclination	42.2	38.7
Right lateral inclination	30.4	31
Left rotation	55.4	59.4
Right rotation	56.9	64.3
**ROM right upper limb**, degrees		
Flexion	152.5	163.8
Extension	59.5	50
Abduction	160.6	180.5
Adduction	45.5	51.7
**ROM left upper limb**, degrees		
Flexion	183.6	182
Extension	48.6	66
Abduction	153.4	143.6
Adduction	40.4	60.8
**ROM right hip**, degrees		
Flexion	86.8	95.3
Extension	43.5	41.8
Abduction	43.9	51.3
Adduction	25	27.3
Internal rotation	53.6	39.4
External rotation	35.9	39
**ROM left hip**, degrees		
Flexion	100.4	115.5
Extension	41.4	32.5
Abduction	39.4	56.4
Adduction	37.8	39.1
Internal rotation	41.7	42.5
External rotation	44.4	55.6

Abbreviations: APA, adapted physical activity; ROM, range of motion.

**Table 2:** Lower limb muscle strength test scores at baseline and post-adapted physical activity protocol.

Variables	Baseline	Post-APA
**Muscle strength**, kilograms		
Right hip flexion	6.5	8.5
Right hip extension	7.1	15.5
Right hip abduction	4.9	7.6
Right hip adduction	6	9.9
Right knee extension	13.5	17.2
Right ankle plantar flexion	5.7	7
Right ankle plantar dorsiflexion	5.5	7
Right ankle inversion	3.4	6.6
Right ankle eversion	2.5	5.9
Left hip flexion	5.5	14.5
Left hip extension	7.8	14.4
Left hip abduction	5.2	11.1
Left hip adduction	5.8	9.4
Left knee extension	8.7	15.9
Left ankle plantar flexion	5.5	7.6
Left ankle plantar dorsiflexion	4.9	7.6
Left ankle inversion	4	7.2
Left ankle eversion	3.8	7.7

Abbreviation: APA, adapted physical activity.

**Table 3:** Measurements of lower limb circumference at several anatomical points at baseline and after physical activity.

Variables	Baseline			Post-APA		
Lower Limb Circumference, mm	Right	Left	Difference	Right	Left	Difference
Groin level	680	680	0	640	645	0.5
Midline of thigh	550	560	1	535	540	0.5
Knee	430	430	0	425	435	1
Half leg	380	380	0	390	395	0.5
Ankle	240	260	2	240	245	0.5
Midline of foot	220	240	2	230	245	1.5

Abbreviation: APA, adapted physical activity.

**Table 4:** Mean scores of quality of life, anxiety/depression status, and distress thermometer questionnaires at baseline and post-adapted physical activity intervention.

Variables	Baseline	Post-APA
**FACT-M**		
Physical wellbeing	27	28
Social/family wellbeing	28	28
Emotional wellbeing	20	21
Functional wellbeing	21	21
Melanoma subscale	59	59
FACT-M total	155	157
Melanoma surgery scale	28	27
**Anxiety/depression status (HADS)**		
Anxiety	2	2
Depression	2	6
**Distress thermometer**	0	0

Abbreviations: APA, adapted physical activity; FACT-M, Functional Assessment of Cancer Therapy–Melanoma; HADS, Hospital Anxiety and Depression Scale.

## Data Availability

All relevant data are included within the manuscript.
